# Homologous MVA and heterologous DREP/MVA vaccine regimens induce robust and durable immune responses against SARS-CoV-2

**DOI:** 10.1038/s41598-026-46699-0

**Published:** 2026-04-09

**Authors:** Patricia Pérez, Gloria Esteso, María A. Noriega, Laura Perez Vidakovics, Peter Liljeström, Gerald M. McInerney, Mariano Esteban, Juan García-Arriaza

**Affiliations:** 1 Department of Molecular and Cellular Biology, Centro Nacional de Biotecnología (CNB), Consejo Superior de Investigaciones Científicas (CSIC), Madrid, Spain; 2 Centro de Investigación Biomédica en Red de Enfermedades Infecciosas (CIBERINFEC), Madrid, Spain; 3 Division of Virology and Immunology, Department of Microbiology, Tumor and Cell Biology, Karolinska Institutet, Stockholm, Sweden

**Keywords:** SARS-CoV-2, DREP, MVA, Immunogenicity, Mice, Immunology, Microbiology

## Abstract

Despite the success of current COVID-19 vaccines, the immunity they generate wanes over time, requiring periodic boosters. The limited durability of memory responses, particularly from mRNA vaccines, remains a major challenge for achieving long-term protection. Developing vaccines that induce more sustained immunity would lessen the need for frequent revaccination, improving global vaccination logistics, especially in resource-limited settings. DNA-launched self-amplifying RNA replicons (DREP) and modified vaccinia virus Ankara (MVA) vectors are promising vaccine platforms capable of inducing potent humoral and cellular immunity. In this study, we evaluated SARS-CoV-2-specific immune responses in C57BL/6 mice following homologous and heterologous prime/boost regimens combining DREP- and MVA-based vaccines expressing the spike (S) protein from either the ancestral Wuhan strain or the Omicron XBB.1.5 variant. Homologous (DREP/DREP, MVA/MVA) and heterologous (DREP/MVA) regimens were followed for six months. MVA-S(3P)-based boosters elicited robust and durable anti-S IgG antibodies cross-recognizing multiple variants, with minimal decay over time. Neutralization mapped to the booster antigen: MVA-S(3P_Wuhan_) induced neutralization of the ancestral strain, while MVA-S(3P_XBB.1.5_) selectively neutralized Omicron subvariants, maintaining high titers for at least six months. MVA-S(3P) boosters also enhanced antibody Fc-effector functions, memory B cells, and T follicular helper responses. Early after boosting, heterologous DREP/MVA regimens induced stronger CD4⁺ and CD8⁺ T-cell responses, while at six months all MVA-S(3P)-boosted groups maintained superior, long-lived cellular immunity. Collectively, MVA-S(3P)-based boosters improved the magnitude, breadth, and durability of humoral and cellular responses, supporting their strategic use in homologous and heterologous DREP/MVA vaccination regimens against SARS-CoV-2 and emerging variants.

**Supplementary Information:**

The online version contains supplementary material available at 10.1038/s41598-026-46699-0.

## Introduction

The unprecedented success of first-generation currently approved COVID-19 vaccines, particularly those based on mRNA platforms, has substantially reduced the burden of severe disease and mortality. However, their protective immunity wanes over time, necessitating repeated booster immunizations^1^. Thus, it is essential to establish vaccination strategies triggering potent and prolonged induction of pathogen-specific B and T cell immune responses. More durable vaccines would lessen the need for frequent revaccination, thereby improving accessibility, reducing logistical and financial constraints, and strengthening pandemic preparedness. This is particularly relevant in vulnerable populations with weaker immune responses, where longer-lasting protection would contribute to better disease control and community-wide immunity.

DNA-launched self-amplifying RNA replicons (DREP) represent an innovative vaccine platform that combines the safety and stability of DNA delivery with the potent immunostimulatory capacity of self-amplifying RNA, eliciting strong adaptive immune responses in preclinical models of viral infection^2–11^ and cancer^12^ models. Previous studies demonstrated that DREP vaccines elicit superior antigen-specific CD8⁺ T-cell and antibody responses compared to conventional plasmid DNA vaccines^6,7,13^. Moreover, recent work from our group comparing DREP-S (expressing a full-length SARS-CoV-2 spike (S) protein with an 18-aa cytoplasmic tail deletion) and DREP-S^ecto^ (expressing a stabilized S ectodomain) showed that DREP-S elicited stronger S-specific humoral and cellular immune responses after a single dose, including high levels of IgG antibodies, high neutralizing antibody titers and robust T-cell activation^11^.

Modified vaccinia virus Ankara (MVA), a highly attenuated poxvirus vector, is another well-established vaccine platform. MVA has shown strong immunogenicity and efficacy in preclinical and clinical studies targeting infectious diseases and cancer, and it is already licensed for use in humans as a smallpox and mpox vaccine^14–16^. MVA is particularly effective at inducing CD8⁺ T-cell responses, which can be further boosted following priming with DNA, mRNA or other viral vectors. Regarding SARS-CoV-2, we previously reported that MVA vectors expressing either the full-length native SARS-CoV-2 S protein (MVA-S) or a prefusion-stabilized S protein (MVA-S(3P)) showed strong humoral and cellular immunogenicity and protective efficacy in mice^17–24^, hamsters^25,26^, and rhesus macaques^27^.

Heterologous prime/boost strategies have demonstrated superior immunogenicity than homologous regimens in several preclinical and clinical studies, potentially offering broader and more durable protection^28^. Indeed, combining DREP and MVA in heterologous prime/boost regimens may leverage the strengths of both platforms to induce potent, long-lasting, and cross-variant immunity, with MVA boosters augmenting the immunogenic potential of DREP. Previous studies from our groups have shown that DREP/MVA combinations yield synergistic effects, enhancing both humoral and cellular immunity against several viruses, such as human immunodeficiency virus (HIV)^6^, chikungunya virus (CHIKV)^3,4,10^, Ebola virus (EBOV)^9^, and hepatitis C virus (HCV)^8^. These findings highlight the potential of DREP/MVA regimens to provide potent and long-lasting protection against SARS-CoV-2 and its evolving variants.

Here, we evaluated in immunized mice the magnitude, breadth, and durability of the SARS-CoV-2-specific humoral and cellular immunogenicity induced by homologous and heterologous vaccination regimens using DREP- and MVA-based vaccine candidates expressing SARS-CoV-2 S proteins from either the ancestral Wuhan strain or the Omicron XBB.1.5 variant. Mice primed with DREP-S or MVA-S and boosted with MVA-S(3P)-based vaccine candidates developed strong and durable anti-S IgG antibodies that remained stable for at least six months and cross-recognized multiple SARS-CoV-2 variants, including Beta, Omicron BA.1, BA.5, XBB.1.5, and XBB.1.16. Boosting with MVA-S(3P_Wuhan_) induced neutralizing antibodies against the ancestral Wuhan strain, while MVA-S(3P_XBB.1.5_) preferentially targeted Omicron subvariants. These boosters also enhanced and sustained different antibody-mediated effector functions. On the cellular level, MVA-S(3P) boosters increased germinal center (GC) B cells, class-switched memory B cells, and Wuhan S-specific T follicular helper (Tfh) cells. Heterologous DREP/MVA regimens elicited stronger early CD4⁺ and CD8⁺ T-cell responses shortly after vaccination than homologous DREP-S/DREP-S or MVA-S/MVA-S(3P) regimens. However, at six months post-boost, all MVA-S(3P)-boosted groups maintained similarly elevated CD4⁺ and CD8⁺ T-cell responses, surpassing those induced by DREP-S/DREP-S. Memory T-cell phenotypes persisted long-term, underscoring the durability of the cellular immune response. Altogether, these results highlight that MVA-based boosters not only amplify immune responses but also enhance their quality and longevity, supporting their strategic use in prime/boost vaccination against SARS-CoV-2 and its variants.

## Results

### Boosting with MVA-S(3P) vaccine candidates induces strong, broad, and durable S-specific IgG responses

**Fig. 1:**
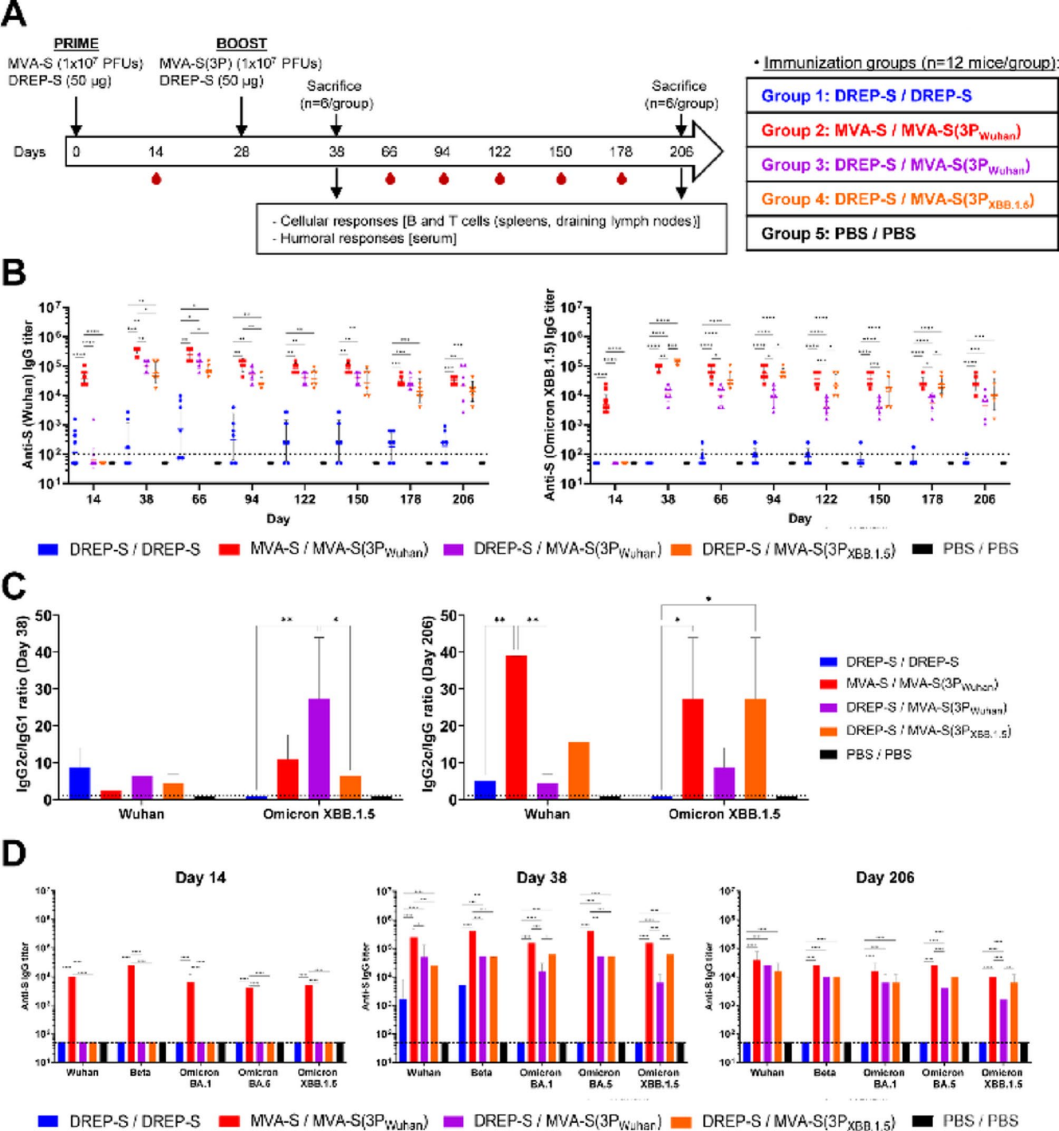
Boosting with MVA-S(3P) vaccine candidates induces strong, cross-reactive, and durable binding antibody responses in C57BL/6 mice. (**A**) Immunization schedule. Female C57BL/6 mice (*n* = 12 per group; 6–8 weeks old) were immunized intramuscularly with a single dose of 50 µg of DREP-S or 1 × 10⁷ PFUs of MVA-S, both expressing the full-length native non-stabilized Wuhan SARS-CoV-2 S protein. Four weeks later, mice received a booster with DREP-S, MVA-S(3P_Wuhan_), or MVA-S(3P_XBB.1.5_) (MVA vectors expressing prefusion-stabilized S proteins from Wuhan or Omicron XBB.1.5, respectively). PBS-inoculated mice served as controls. At day 38 (10 days post-boost), half of the mice (*n* = 6 per group) were euthanized and serum and spleens from each mouse were collected to evaluate SARS-CoV-2-specific humoral and cellular immune responses. The remaining animals (*n* = 6 per group) were bled monthly (at days 66, 94, 122, 150, and 178) and sacrificed on day 206 (six months post-boost) where serum and spleens were also obtained. Data shown are derived from a single independent in vivo experiment. (**B**) Longitudinal analysis of total anti-S IgG binding titers against Wuhan and Omicron XBB.1.5 proteins. Data measured by ELISA, in duplicate, in individual mouse serum samples collected on days 14, 38, 66, 94, 122, 150, 178, and 206. Geometric means ± standard deviations (SD) are shown. Dotted line indicates the limit of detection. Mixed-effects model with Geisser-Greenhouse correction followed by Tukey’s multiple comparisons test of transformed data: *p < 0.033; **p < 0.002; ***p < 0.0002; ****p < 0.0001. (**C**) IgG2c/IgG1 isotype ratios against Wuhan and XBB.1.5 S proteins in pooled sera of each group from days 38 (left panel) and 206 (right panel). Anti-S IgG1 and IgG2c titers against Wuhan and Omicron XBB.1.5 S proteins were determined by ELISA, in duplicate, and the IgG2c/IgG1 ratio of endpoint titers for each group are represented. Ordinary two-way ANOVA followed by Tukey’s multiple comparisons test: *p < 0.033; **p < 0.002. (**D**) Cross-reactive anti-S IgG titers against proteins from Wuhan, Beta, Omicron BA.1, BA.5, and XBB.1.5. Data measured by ELISA, in duplicate, in pooled sera of each group on days 14, 38 and 206. Geometric means ± SD of endpoint titers for each group are represented. Dotted line represents the limit of detection. Ordinary two-way ANOVA followed by Tukey’s multiple comparisons test of transformed data: *p < 0.033; **p < 0.002; ***p < 0.0002; ****p < 0.0001.

To evaluate the magnitude, breadth, and durability of antibody responses against SARS-CoV-2 induced by DREP- and MVA-based vaccine candidates, C57BL/6 mice (*n* = 12/group) were immunized intramuscularly with DREP-S or MVA-S, both encoding the ancestral Wuhan SARS-CoV-2 native S protein (Fig. 1A). Mice were bled two weeks later, and at week 4 received a booster with DREP-S, MVA-S(3P_Wuhan_), or MVA-S(3P_XBB.1.5_) (both MVA-S(3P) vaccine candidates express a prefusion-stabilized S protein from Wuhan or Omicron XBB.1.5, respectively), resulting in homologous (DREP-S/DREP-S, MVA-S/MVA-S(3P)) or heterologous (DREP-S/MVA-S(3P_Wuhan_) and DREP-S/MVA-S(3P_XBB.1.5_)) vaccination regimens (Fig. 1A). Control mice received PBS. On day 38 (10 days post-boost), half of the mice (*n* = 6/group) were sacrificed, and serum samples, draining lymph nodes, and spleens were collected to evaluate SARS-CoV-2-specific humoral and T-cellular immune responses, respectively. The remaining animals (*n* = 6/group) were bled monthly (at days 66, 94, 122, 150, and 178) and finally sacrificed on day 206 (six months post-boost) where serum samples and spleens were similarly obtained.

To evaluate SARS-CoV-2-specific humoral responses, S-specific serum IgG antibody titers were determined by ELISA at multiple time points (Fig. 1B). Following priming (day 14), MVA-S induced substantially higher anti-S IgG titers than DREP-S against both Wuhan and Omicron XBB.1.5 variants. Geometric mean titers in MVA-S-immunized mice reached 3.93 × 10⁴ (Wuhan) and 5.33 × 10³ (Omicron XBB.1.5), whereas DREP-S elicited only low titers against Wuhan (1.35 × 10²) and none against XBB.1.5 (Fig. 1B). Following the booster (day 28), all regimens boosted with MVA-S(3P)-based vaccine candidates (MVA-S(3P_Wuhan_) or MVA-S(3P_XBB.1.5_)) elicited marked increases in anti-S IgG titers, peaking between days 38 and 66 (days 10 and 38 post-boost). The highest anti-S(Wuhan) IgG titers were observed in the MVA-S/MVA-S(3P_Wuhan_) group (3.43 × 10⁵ at day 38) (Fig. 1B, left panel), while the highest anti-S(XBB.1.5) IgG titers were detected in the DREP-S/MVA-S(3P_XBB.1.5_) group (1.35 × 10⁵ at day 38) (Fig. 1B, right panel). Notably, anti-S IgG antibody titers remained stable up to six months post-boost (day 206), with less than 35% decline in all MVA-S(3P)-boosted groups (Fig. 1B). In particular, the MVA-S/MVA-S(3P_Wuhan_) vaccination group showed a 9.1% and 30.2% reduction in anti-S IgG antibody titers against Wuhan and Omicron XBB.1.5, respectively; DREP-S/MVA-S(3P_Wuhan_) a reduction of 18.2% and 34.3%, and DREP-S/MVA-S(3P_XBB.1.5_) a 18.7% and 7.91% reduction (Fig. 1B). On the other hand, anti-S IgG antibody titers against Wuhan induced by DREP-S/DREP-S persisted over time but remained significantly lower than in the other groups and showed high inter-animal variability (Fig. 1B, left panel).

To further characterize the type of antibody response against SARS-CoV-2, we next measured the anti-S IgG2c and IgG1 antibody titers against Wuhan and Omicron XBB.1.5, and calculated the corresponding IgG2c/IgG1 ratio in pooled sera from days 38 and 206 (10 days and 6 months post-boost, respectively), as a surrogate of Th1/Th2 bias (Fig. 1C). All vaccination regimens induced a Th1-skewed response (IgG2c/IgG1 > 1), consistent with protective immunity. Remarkably, this IgG2c/IgG1 ratio further increased over time in the MVA-S/MVA-S(3P_Wuhan_) and DREP-S/MVA-S(3P_XBB.1.5_) groups, suggesting maturation of the Th1-biased response (Fig. 1C).

Next, we assessed the breadth of the SARS-CoV-2-specific humoral response by measuring by ELISA in pooled mouse sera anti-S IgG antibody titers binding to multiple SARS-CoV-2 variants (Beta, Omicron BA.1, BA.5, and XBB.1.5). After priming (day 14), only MVA-S-primed mice displayed detectable anti-S IgG titers across variants, comparable to those against Wuhan S (Fig. 1D, left panel). By day 38 (10 days post-boost), all groups boosted with MVA-S(3P)-based vaccine candidates exhibited strong cross-reactive anti-S IgG titers across all variants. The MVA-S/MVA-S(3P_Wuhan_) group consistently showed the highest anti-S IgG titers, with all titers being of the same order of magnitude (between 10^5^ and 10^6^) and comparable to those obtained against the Wuhan S (Fig. 1D, middle panel). Interestingly, DREP-S/MVA-S(3P_XBB.1.5_) induced higher anti-S IgG titers against Omicron BA.1 and XBB.1.5 than DREP-S/MVA-S(3P_Wuhan_), while both groups induced similar titers against Beta and Omicron BA.5 (Fig. 1D, middle panel). In contrast, DREP-S/DREP-S induced only modest anti-S IgG titers against Wuhan and Beta (between 10^3^ and 10^4^) and failed to induce detectable responses to Omicron variants (Fig. 1D, middle panel). At day 206 (6 months post-boost), durable anti-S IgG titers (10³–10⁵) against all variants were maintained only in groups boosted with MVA-S(3P)-based vaccine candidates (Fig. 1D, right panel).

### MVA-S(3P) boosters induce broad and durable neutralizing antibody responses

**Fig. 2:**
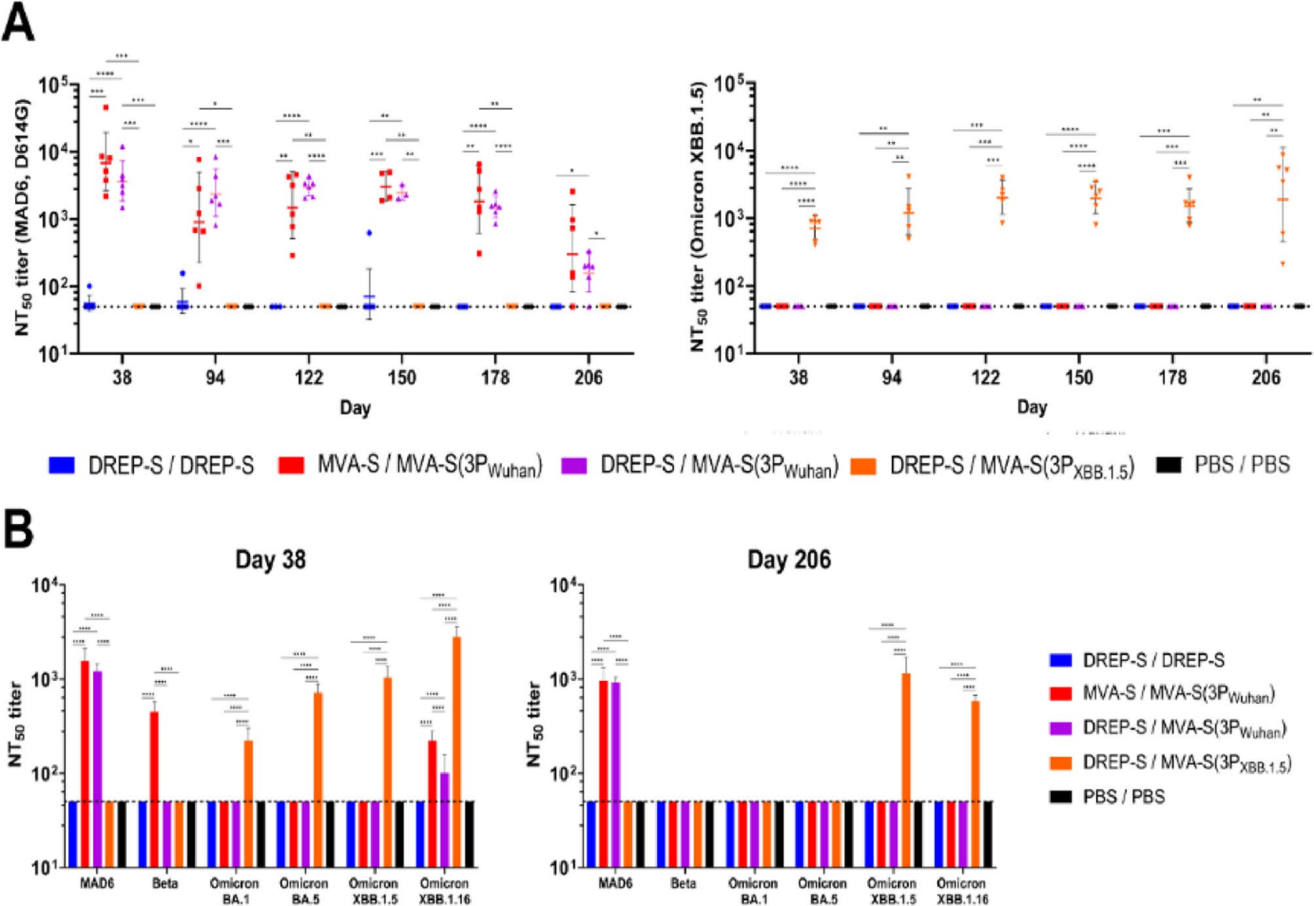
MVA-S(3P)-based boosters induce broad and durable neutralizing antibody responses in immunized mice. (**A**) Longitudinal analysis of neutralizing antibody titers (NT_50_) against SARS-CoV-2 Wuhan (MAD6 isolate) and Omicron XBB.1.5. NT_50_ titers from individual serum samples collected at days 38, 94, 122, 150, 178, and 206 were measured in triplicate by a live virus microneutralization (MNT) assay. Geometric means ± SD of NT_50_ values of each sample for each group are shown. Dotted line indicates the limit of detection. Mixed-effects model with the Geisser-Greenhouse correction followed by Tukey’s multiple comparisons test of transformed data: *p < 0.033; **p < 0.002; ***p < 0.0002; ****p < 0.0001. (**B**) Cross-neutralization of SARS-CoV-2 Wuhan, Beta, Omicron BA.1, BA.5, XBB.1.5, and XBB.1.16. NT_50_ titers from pooled sera of each group collected at days 38 and 206 were analyzed in triplicate by a live virus MNT assay. Geometric means ± SD of NT_50_ titers of each pooled group sample are shown. Dotted line represents the limit of detection. Ordinary two-way ANOVA followed by Tukey’s multiple comparisons test of transformed data: ****p < 0.0001.

To assess the functional quality of vaccine-induced antibodies, we performed live-virus neutralization assays with sera collected at multiple time points post-boost (days 38 to 206; 10 days to 6 months post-boost, respectively). Neutralizing activity was initially measured against the ancestral SARS-CoV-2 Wuhan strain (MAD6 isolate) and the Omicron XBB.1.5 subvariant in individual serum samples (Fig. 2A).

Neutralization of Wuhan MAD6 was observed only in groups boosted with MVA-S(3P_Wuhan_) (Fig. 2A, left panel). MVA-S/MVA-S(3P_Wuhan_) and DREP-S/MVA-S(3P_Wuhan_) groups reached peak NT₅₀ titers of 7.12 × 10³ and 3.66 × 10³ at day 38, respectively, followed by a gradual decline over time. By day 206, neutralization capacity remained detectable in both groups (3.54 × 10² and 1.63 × 10², respectively) (Fig. 2A, left panel). In contrast, neutralization of Omicron XBB.1.5 was restricted to MVA-S(3P_XBB.1.5_)-boosted mice (Fig. 2A, right panel). The DREP-S/MVA-S(3P_XBB.1.5_) regimen elicited stable NT₅₀ titers of 1–2 × 10³ across the six-month follow-up, demonstrating enhanced durability and specificity toward this highly immune-evasive variant (Fig. 2A, right panel). The DREP-S/DREP-S regimen failed to induce neutralizing antibodies against either Wuhan MAD6 or Omicron XBB.1.5 at any time point (Fig. 2A).

We next evaluated neutralization breadth using pooled sera from days 38 and 206 against the ancestral SARS-CoV-2 Wuhan MAD6 and additional SARS-CoV-2 variants of concern (Beta, Omicron BA.1, BA.5, XBB.1.5, and XBB.1.16) (Fig. 2B). At both time points, only vaccine regimens boosted with MVA-S(3P)-based vaccine candidates retained detectable neutralizing activity. At day 38, sera from mice vaccinated with MVA-S/MVA-S(3P_Wuhan_) neutralized Wuhan MAD6, Beta (being the only group showing detectable NT_50_ titers), and Omicron XBB.1.16, whereas sera from mice vaccinated with DREP-S/MVA-S(3P_Wuhan_) only neutralized Wuhan MAD6 and Omicron XBB.1.16. In contrast, sera from mice vaccinated with DREP-S/MVA-S(3P_XBB.1.5_) neutralized all Omicron subvariants, with the highest NT_50_ titers recorded against XBB.1.16 (Fig. 2B, left panel). By day 206, neutralization breadth had narrowed: MVA-S/MVA-S(3P_Wuhan_) and DREP-S/MVA-S(3P_Wuhan_) sera retained activity only against Wuhan MAD6 and did not exhibit detectable NT_50_ titers against any of the tested variants, while sera from the DREP-S/MVA-S(3P_XBB.1.5_) uniquely neutralized Omicron XBB.1.5 and XBB.1.16 subvariants. No vaccination group showed detectable NT_50_ titers against Beta, Omicron BA.1 or BA.5 at this time point (Fig. 2B, right panel). The DREP-S/DREP-S regimen showed no detectable neutralizing activity against any variant at either day 38 or day 206 (Fig. 2B).

### MVA-S(3P) boosters enhance and sustain antibody-mediated effector functions

In addition to virus neutralization, we evaluated antibody-mediated Fc effector functions, including antibody-dependent cellular phagocytosis (ADCP), antibody-dependent complement deposition (ADCD), and antibody-dependent natural killer (NK) cell activation (ADNKA) in serum samples obtained at days 38 and 206 (10 days and 6 months post-boost, respectively) from vaccinated mice.

**Fig. 3:**
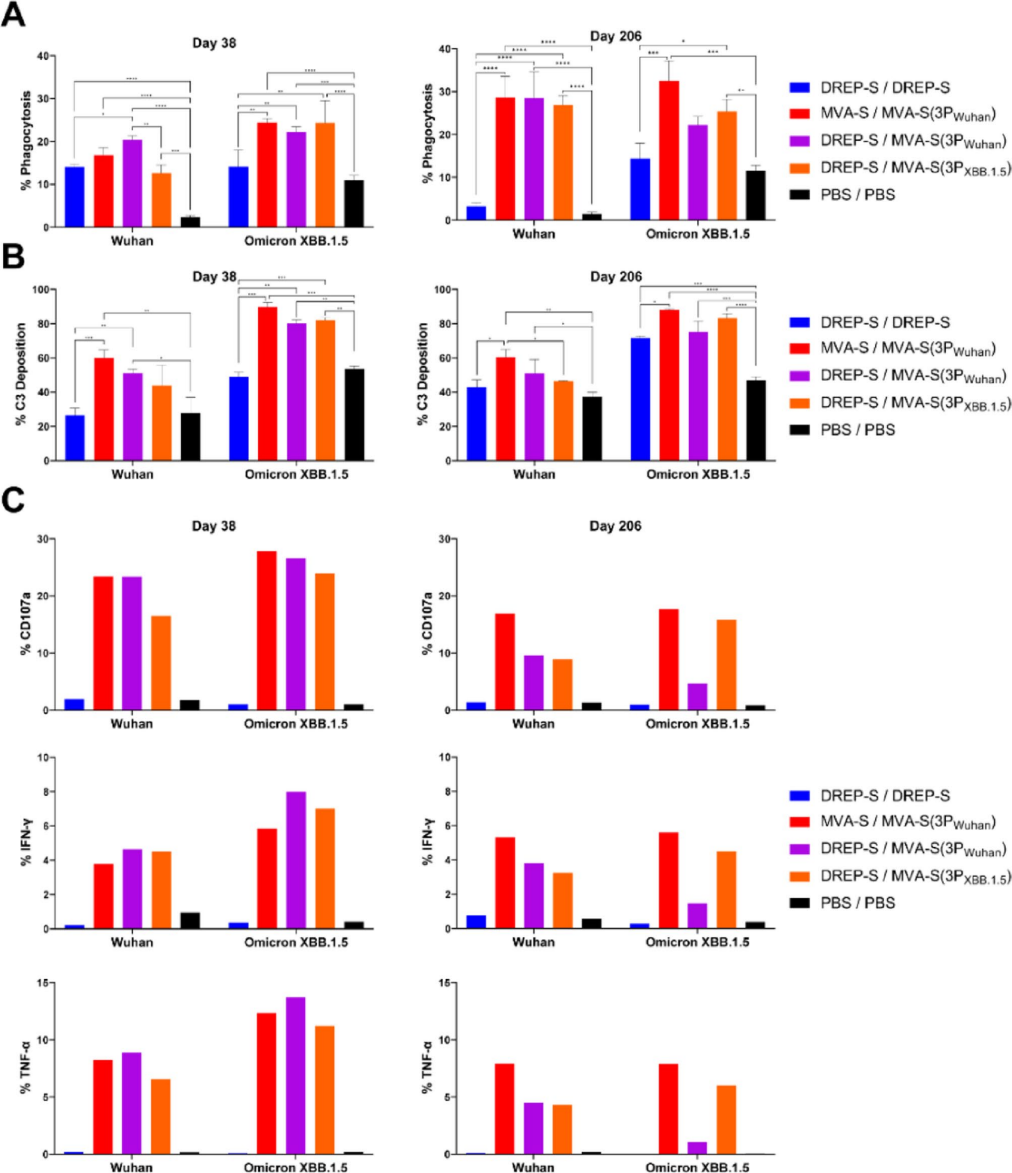
MVA-S(3P)-based boosters develop robust and durable Fc-dependent antibody effector functions in immunized mice. (**A**) ADCP activity against fluorescent beads coated with SARS-CoV-2 S proteins from Wuhan and XBB.1.5. Data from pooled sera of each group collected on day 38 (left panel) and day 206 (right panel) were measured in triplicate and are represented as the mean ± SD of phagocytosis percentage. Ordinary two-way ANOVA followed by Tukey’s multiple comparisons test: *p < 0.033; **p < 0.002; ***p < 0.0002; ****p < 0.0001. (**B**) ADCD activity against fluorescent beads coated with SARS-CoV-2 S proteins from Wuhan and XBB.1.5. Data from pooled sera of each group collected on day 38 (left panel) and day 206 (right panel) were measured in triplicate and are presented as the mean ± SD of complement C3 deposition percentage. Ordinary two-way ANOVA followed by Tukey’s multiple comparisons test: *p < 0.033; **p < 0.002; ***p < 0.0002; ****p < 0.0001. (**C**) ADNKA against SARS-CoV-2 S proteins from Wuhan and XBB.1.5. Data from pooled sera of each group collected on day 38 (left panel) and day 206 (right panel) represent the percentages of NK cells expressing CD107a degranulation marker (upper panels), secreting IFN-γ (middle panels) or TNF-α (lower panels).

In the ADCP assay, all vaccination groups exhibited robust serum-mediated phagocytosis against Wuhan S-coated beads at day 38, with the percentage of phagocytosis being at least fourfold higher than those observed in sera from the PBS control group. The highest phagocytic activity was observed in the DREP-S/MVA-S(3P_Wuhan_) and MVA-S/MVA-S(3P_Wuhan_) groups (Fig. 3A, left panel). Notably, only MVA-S(3P)-boosted groups showed significant phagocytic activity against Omicron XBB.1.5 S-coated beads, compared to the PBS control group (Fig. 3A, left panel). By day 206, all regimens including MVA-S(3P) as a booster dose exhibited even higher phagocytic activity against both Wuhan and Omicron XBB.1.5 S-coated beads, suggesting antibody maturation and functional specialization (Fig. 3A, right panel).

Regarding ADCD, at day 38 only MVA-S/MVA-S(3P_Wuhan_) and DREP-S/MVA-S(3P_Wuhan_) groups induced significant serum-mediated complement deposition against Wuhan S-coated beads compared to the PBS control group or the DREP-S/DREP-S regimen. In contrast, all MVA-S(3P)-boosted groups showed higher complement deposition against Omicron XBB.1.5 S-coated beads than the PBS control group or the DREP-S/DREP-S regimen (Fig. 3B, left panel). These patterns of complement deposition activity were maintained through day 206 with comparable magnitudes (Fig. 3B, right panel).

In ADNKA assays, at day 38 all MVA-S(3P)-boosted groups elicited increased serum-mediated NK cell activation against Wuhan and Omicron XBB.1.5 S-coated beads, reflected by higher expression levels of CD107a (a degranulation marker), IFN-γ, and TNF-α by NK cells, compared with the PBS control group and the DREP-S/DREP-S regimen (Fig. 3C, left panels). By day 206 all groups showed a reduced capacity of serum samples to activate NK cells though the MVA-S/MVA-S(3P_Wuhan_) group maintained the highest levels of NK cells expressing CD107a, IFN-γ, and TNF-α against Wuhan and Omicron XBB.1.5 S-coated beads (Fig. 3C, right panels).

### MVA-S(3P) boosters increase GC B cells and class-switched memory B cells

**Fig. 4:**
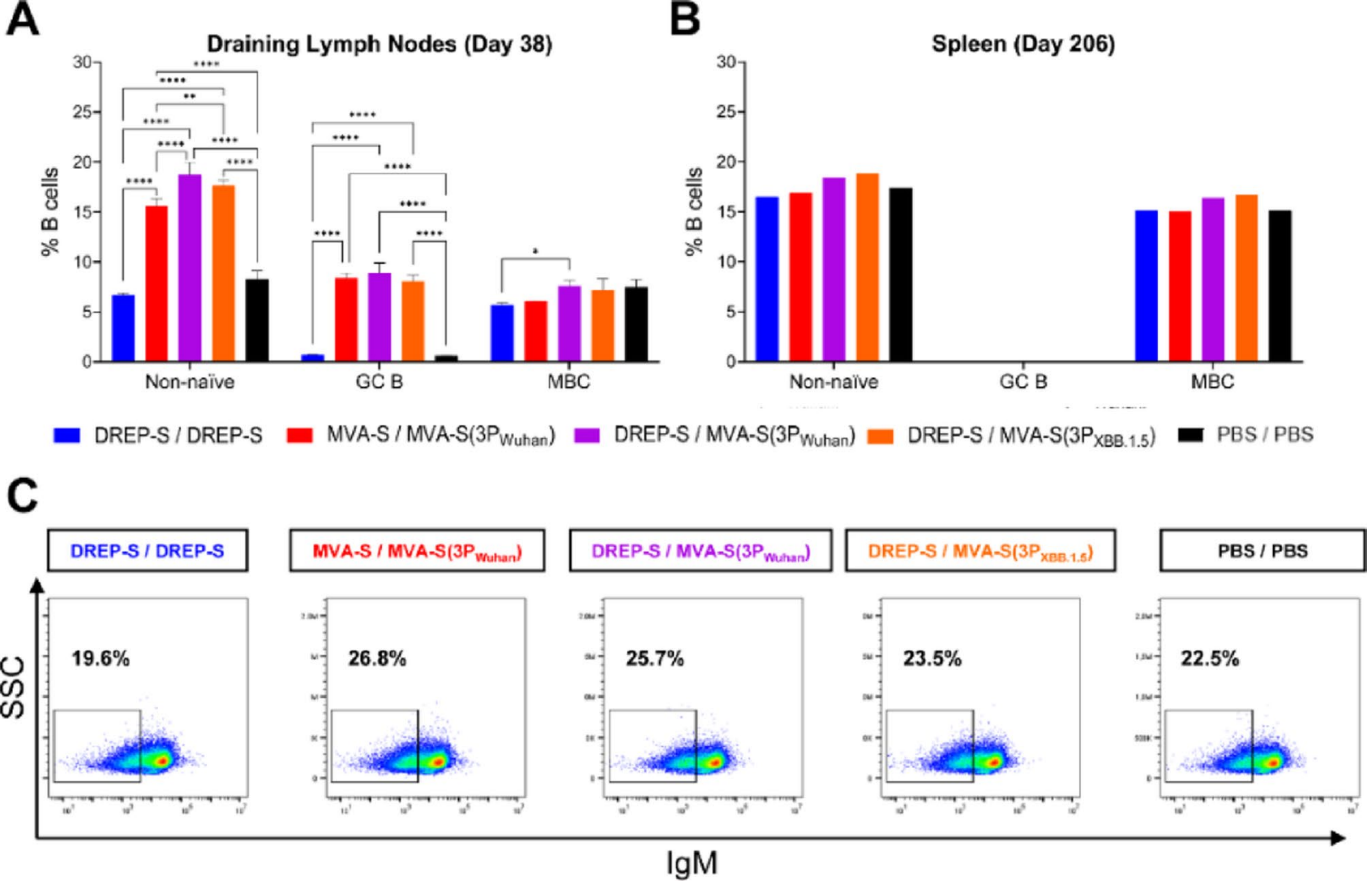
MVA-S(3P)-based boosters favor GC and class-switched memory B-cell responses in immunized mice. (**A**) Percentages (mean ± SD) of B cells (B220⁺CD19⁺) that were IgD⁻ (Non-naïve B cells), GL7⁺CD38⁻ (GC B cells), and GL7⁻CD38⁺ (MBC), analyzed by flow cytometry in technical triplicates of pooled draining (inguinal) lymph node samples collected on day 38. Ordinary two-way ANOVA followed by Tukey’s multiple comparisons test: *p < 0.033; **p < 0.002; ****p < 0.0001. (**B**) Percentages (mean ± SD) of B cells (B220⁺CD19⁺) that were IgD⁻ (activated or memory B cells), GL7⁺CD38⁻ (GC B cells), and GL7⁻CD38⁺ (MBC), analyzed by flow cytometry in pooled spleen samples collected on day 206. (**C**) Representative flow cytometry plots and frequencies of IgD^−^IgM^−^ swMBCs in each immunization group.

Because B-cell phenotyping informs the magnitude and quality of antibody responses, we next profiled draining lymph nodes obtained from immunized mice on day 38 (10 days post-boost) by flow cytometry to quantify GC B cells and memory B cells (MBCs). B220⁺CD19⁺ B cells lacking IgD (non-naïve) were gated and further subdivided into GC B cells (GL7^+^CD38^−^) and MBCs (GL7^−^CD38^+^) subsets. The results showed that MVA-S(3P)-boosted groups showed higher frequencies of non-naïve and GC B cells than homologous DREP-S/DREP-S and PBS control group (Fig. 4A). No differences in MBC frequencies were observed across vaccination regimens relative to the PBS control group.

At six months post-boost (day 206), spleens from immunized mice were analysed to quantify the same B-cell subsets assessed at day 38. The spleen, a reservoir for MBCs, is well suited for long-term evaluation of B-cell phenotypes and sustained humoral responses. In addition, IgM expression was also measured to identify IgD⁻IgM⁻ isotype-switched MBCs (swMBCs), which have undergone affinity maturation and whose persistence is a key indicator of durable, high-quality humoral immunity. The analysis showed that overall MBC frequencies were similar across vaccinated groups and PBS control group (Fig. 4B), but mice boosted with MVA-S(3P) vaccine candidates displayed slightly higher frequencies of swMBCs than DREP-S/DREP-S and PBS control group (Fig. 4C).

### MVA-S(3P) boosters promote expansion and functional maturation of CD4⁺ Tfh responses

**Fig. 5:**
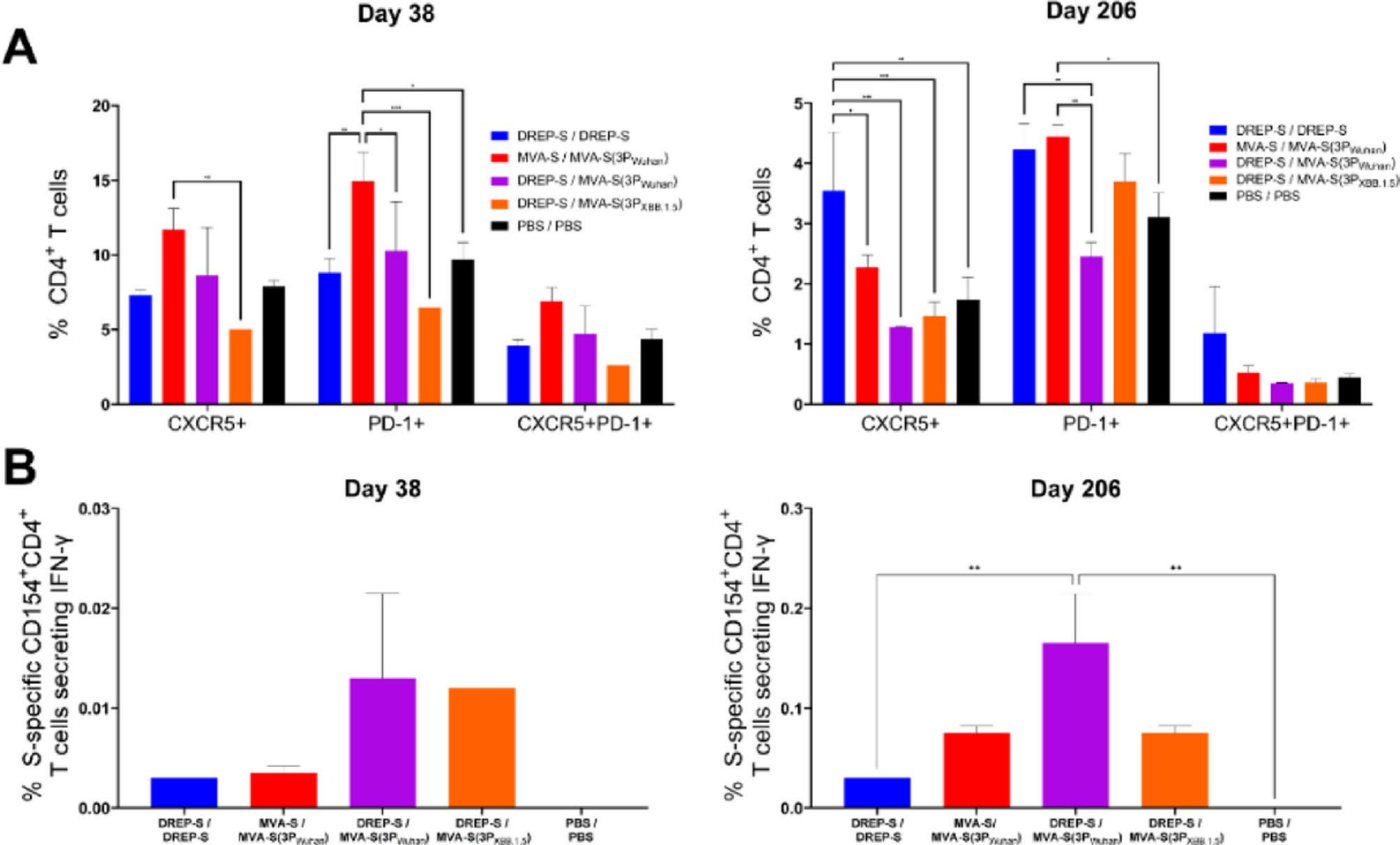
Boosting with Wuhan S-expressing vaccines promotes expansion and maturation of S-specific CD4^+^ Tfh cells in immunized mice. (**A**) Percentages (mean ± SD) of SARS-CoV-2 S-specific CD4⁺ T cells expressing CXCR5, PD-1 or both (Tfh cells) at days 38 and 206, analyzed in pooled spleen samples. Ordinary two-way ANOVA followed by Tukey’s multiple comparisons test: *p < 0.033; **p < 0.002; ***p < 0.0002. (**B**) Percentages (mean ± SD) of SARS-CoV-2 S-specific CD4⁺ Tfh cells co-expressing CD154 (CD40L) and IFN-γ at days 38 and 206, analyzed in pooled spleen samples. Ordinary one-way ANOVA followed by Tukey’s multiple comparisons test: **p < 0.002.

Since CD4⁺ Tfh cells are key orchestrators of humoral immunity, we next evaluated their induction following vaccination. Therefore, splenocytes from immunized mice were re-stimulated with SARS-CoV-2 Wuhan S protein plus a Wuhan S peptide pool, and the frequencies of CD4⁺ T cells expressing CXCR5, PD-1, or both surface markers were assessed. At day 38 (10 days post-boost), the MVA-S/MVA-S(3P_Wuhan_) group showed the highest frequencies of CXCR5^+^, PD-1^+^, and CXCR5⁺PD-1⁺ CD4⁺ T cells, all above PBS control, indicating robust expansion of the CD4⁺ Tfh cell compartment (Fig. 5A, left panel). By day 206 (six months post-boost), frequencies of CXCR5^+^, PD-1^+^, and CXCR5⁺PD-1⁺ CD4⁺ T cells had declined across all groups, but the frequencies of CXCR5⁺ or PD-1⁺ CD4⁺ T cells in the DREP-S/DREP-S and MVA-S/MVA-S(3P_Wuhan_) groups remained higher than those observed in the PBS control group (Fig. 5A, right panel).

Next, to assess Tfh functionality, we analyzed co-expression of CD154 (CD40L) and IFN-γ, markers of activated Th1-like Tfh cells. At day 38 (10 days post-boost), Wuhan S-specific CD4⁺CD154⁺IFN-γ⁺ Tfh cells were virtually undetectable in all groups (see low scale values in Fig. 5B, left panel). However, by day 206 (six months post-boost), their frequencies increased modestly, with the highest levels observed in the DREP-S/MVA-S(3P_Wuhan_) group, followed by MVA-S/MVA-S(3P_Wuhan_) and DREP-S/MVA-S(3P_XBB.1.5_) groups (ten-fold scale in Fig. 5B, right panel), suggesting maturation of Tfh memory quality over time.

### MVA-S(3P) boosters enhance the magnitude, polyfunctionality, and persistence of S-specific CD4⁺ and CD8⁺ T-cell responses

To investigate the T cell-mediated immune responses induced by the different vaccination regimens, an intracellular cytokine staining (ICS) assay was performed on splenocytes isolated at day 38 (10 days post-boost) and day 206 (6 months post-boost). Cells were stimulated ex vivo with peptide pools spanning the full-length Wuhan SARS-CoV-2 S protein, and expression of CD107a (a degranulation marker), and/or secretion of IFN-γ, TNF-α, and IL-2 were measured in both CD4⁺ and CD8⁺ T cells.

**Fig. 6:**
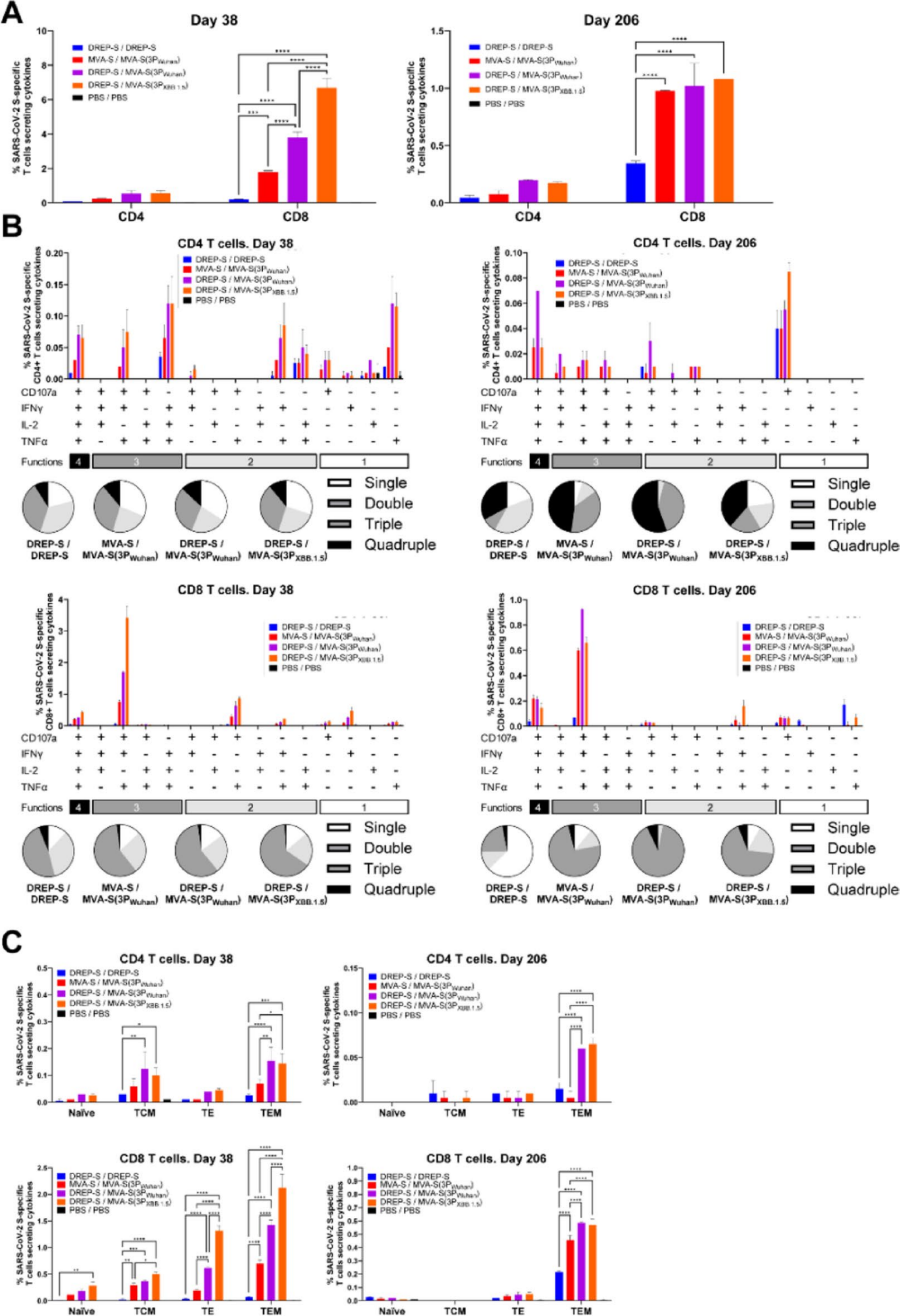
MVA-S(3P)-based boosters enhance the magnitude, polyfunctionality, and persistence of S-specific CD4⁺ and CD8⁺ T-cell responses in immunized mice. SARS-CoV-2 S-specific T-cell immune responses, directed against Wuhan S peptide pools, were analyzed in pooled splenocytes from each group obtained at days 38 (left panels) and 206 (right panels), and evaluated by ICS, as described in Materials and Methods. (**A**) Magnitude of S-specific CD4^+^ and CD8^+^ T-cell responses in pooled splenocytes harvested at days 38 (left panel) and 206 (right panel). Percentages (mean ± SD) of CD4^+^ or CD8^+^ T cells expressing CD107a and/or producing IFN-γ and/or TNF-α and/or IL-2 are shown. Data are from duplicates of each pooled group sample. Ordinary two-way ANOVA followed by Tukey’s multiple comparisons test: ***p < 0.0002; ****p < 0.0001. (**B**) Polyfunctional profiles of S-specific CD4^+^ and CD8^+^ T-cell responses in pooled splenocytes harvested at days 38 (left panel) and 206 (right panel). Frequencies of S-specific CD4^+^ and CD8^+^ T cells simultaneously expressing 1 to 4 functions (CD107a, IFN-γ, TNF-α, IL-2) are shown. Pie charts show the distribution of single-, double-, triple-, and quadruple-function subsets (should be corrected for the corresponding samples in the Figure). (**C**) Memory phenotypes of S-specific CD4^+^ and CD8^+^ T-cell responses in pooled splenocytes harvested at days 38 (left panel) and 206 (right panel). Percentages of naive (CD127^−^/CD62L^+^), T central memory (TCM, CD127^+^/CD62L^+^), T effector memory (TEM; CD127^+^/CD62L^−^), and T effector (TE; CD127^−^/CD62L^−^) CD4^+^ and CD8^+^ T cells expressing CD107a, and/or secreting IFN-γ, TNF-α, and IL-2 in response to stimulation with S peptide pools are shown. Ordinary two-way ANOVA followed by Tukey’s multiple comparisons test: *p < 0.033; **p < 0.002; ***p < 0.0002; ****p < 0.0001.

At both time points, S-specific CD4⁺ and CD8⁺ T cells were detected in all vaccinated groups, with CD8⁺ T cells dominating the response, and frequencies decreasing approximately five-fold between day 38 and day 206. All MVA-S(3P)-boosted groups induced significantly higher frequencies of S-specific CD4⁺ and CD8⁺ T-cell responses than homologous DREP-S/DREP-S group (Fig. 6A). On day 38, the strongest S-specific CD4⁺ and CD8⁺ T-cell responses were observed in the DREP-S/MVA-S(3P_XBB.1.5_) group (0.56% CD4⁺ T cells and 6.69% CD8⁺ T cells), followed by DREP-S/MVA-S(3P_Wuhan_) (0.55% CD4⁺ T cells and 3.81% CD8⁺ T cells). MVA-S/MVA-S(3P_Wuhan_) also induced strong S-specific CD4⁺ and CD8⁺ T-cell responses (0.26% CD4⁺ T cells and 1.81% CD8⁺ T cells), whereas DREP-S/DREP-S was largely ineffective (0.10% CD4⁺ T cells and 0.21% CD8⁺ T cells), highlighting the poor performance under intramuscular administration of the homologous DREP-S/DREP-S regimen in eliciting cellular immunity (Fig. 6A, left panel). By day 206, S-specific CD4⁺ and CD8^+^ T-cell responses had decreased in magnitude in all vaccinated groups. Regarding S-specific CD4⁺ T-cell responses the magnitude was below 0.2% in all vaccinated groups. In contrast, for S-specific CD8⁺ T-cell responses, despite the decrease in magnitude, S-specific CD8⁺ T-cell frequencies remained higher in all MVA-S(3P)-boosted regimens (0.98%-1.08%) compared with homologous DREP-S/DREP-S regimen (0.35%) (Fig. 6A, right panel).

We next assessed the polyfunctionality of the S-specific CD4⁺ and CD8^+^ T-cell responses by evaluating the expression of CD107a, and/or secretion of IFN-γ, TNF-α, and IL-2. On day 38, all MVA-S(3P)-boosted groups elicited highly polyfunctional S-specific CD4⁺ and CD8⁺ T-cell responses, with > 66% and > 85% of S-specific CD4⁺ and CD8⁺ T cells, respectively, expressing two or more functions (Fig. 6B, left panels, pie charts). The predominant S-specific CD4⁺ T-cell subset in all groups secreted IFN-γ, TNF-α, and IL-2 (Fig. 6B, upper left panel, graph), while S-specific CD8⁺ T cells most frequently secreted IFN-γ and TNF-α and expressed CD107a (Fig. 6B, bottom left panel, graph). At day 206, polyfunctionality was further enhanced in nearly all vaccination groups: >77% and > 89% of S-specific CD4⁺ and CD8⁺ T cells, respectively, retained two or more functions (Fig. 6B, right panels, pie charts), except in the DREP-S/DREP-S regimen (< 50% of S-specific CD8⁺ T cells) (Fig. 6B, bottom right panel, pie chart). At this later time, S-specific CD4⁺ T cells were mainly CD107a^+^ in nearly all groups, although this population represented less than 50% of the total S-specific CD4⁺ T cells (Fig. 6B, upper right panel, graph). An exception was the DREP-S/MVA-S(3P_Wuhan_) group, which showed the highest frequency of S-specific CD4⁺ T cells exhibiting all four functions (Fig. 6B, upper right panel, graph). For S-specific CD8⁺ T cells, the IFN-γ⁺TNF-α⁺CD107a⁺ subset remained predominant across groups, as also observed on day 38 (Fig. 6B, bottom right panel, graph).

Memory phenotyping of S-specific CD4⁺ and CD8⁺ T cells was determined using CD62L (which facilitates the migration of T cells to the lymph nodes for a primary immune response) and CD127 (IL-7 receptor, which allows T cells to receive signals to remain in circulation during immunological memory and is crucial for long-term survival) as markers. At day 38, the majority of S-specific CD4⁺ T-cell subpopulations in all vaccinated groups were T effector memory (TEM: CD62L⁺CD127⁻) and T central memory (TCM: CD62L⁺CD127⁺) subsets, while S-specific CD8⁺ T cells in all vaccinated groups were predominantly TEM (Fig. 6C, left panels). By day 206, the majority of both S-specific CD4⁺ and CD8⁺ T-cell subpopulations in all vaccinated groups were almost exclusively TEM (Fig. 6C, right panels).

## Discussion

The emergence of immune-evasive SARS-CoV-2 variants and the waning immunity observed after current COVID-19 vaccination campaigns underscore the urgent need for vaccine strategies capable of eliciting potent, broad, and durable protective immune responses^29,30^. Heterologous prime/boost vaccination regimens have gained attention for their capacity to synergistically activate multiple arms of the immune system, often surpassing the immunogenicity induced by homologous approaches. Indeed, combinations of SARS-CoV-2 vaccines, including mRNA, viral vectors, and protein subunits, have consistently induced stronger and more protective humoral and cellular immune responses than the homologous counterpart^31–33^. The heterologous findings highlight the potential of prime/boost strategies as effective vaccination approaches against SARS-CoV-2 and other pathogens.

Our collaborative groups have previously validated DREP/MVA heterologous vaccination for improved immunogenicity and efficacy against several viral infectious diseases^2–11^. For example, DREP/MVA vaccination induced robust immune responses and high protection against CHIKV in mice and non-human primates^3,4,10^, as well as strong CD8⁺ T-cell responses against Ebolavirus^9^. In the context of HCV, DREP/MVA vaccination enhanced both antibody and CD8⁺ T-cell responses compared to homologous DREP/DREP or MVA/MVA regimens^8^, with similar advantages reported for HIV^6^. Collectively, these studies demonstrate the versatility and effectiveness of DREP/MVA regimens across diverse pathogens.

With the aim to investigate how the immune system can be programmed through vaccination with non-viral and viral vectors, we have analyzed in depth in a mouse model the contribution of DREP and MVA vectors, alone or in combination, for their ability to trigger broad and long-term immune responses. Thus, in this study, we evaluated SARS-CoV-2-specific humoral and cellular immunity in C57BL/6 mice following homologous or heterologous prime/boost regimens using DREP- and MVA-based vaccines expressing the full-length S protein from either the ancestral Wuhan strain (DREP-S, MVA-S, and the prefusion-stabilized MVA-S(3P_Wuhan_)) or the Omicron XBB.1.5 variant (MVA-S(3P_XBB.1.5_)). To date, anti-SARS-CoV-2 antibody titers remain the main correlate of protection^34^. In this study, we found that shortly after boosting, MVA-S/MVA-S(3P_Wuhan_) regimen induced slightly higher binding IgG antibody titers against the Wuhan S protein than the DREP-S/MVA-S(3P_Wuhan_) regimen. Strikingly, despite not encoding the Omicron XBB.1.5 S protein, MVA-S/MVA-S(3P_Wuhan_) regimen also elicited high IgG antibody titers against Omicron XBB.1.5 S, comparable to those induced by DREP-S/MVA-S(3P_XBB.1.5_), which does encode this variant S. Additionally, those binding anti-S IgG responses were Th1-skewed, with higher levels of anti-S IgG2c versus IgG1 antibody titers. Importantly, binding anti-S IgG antibody titers in the MVA-S(3P)-boosted groups recognized different SARS-CoV-2 variants (Beta, Omicron BA.1, BA.5, XBB.1.5, and XBB.1.16) and declined only slightly over six months, underscoring the breadth and durability of immune responses elicited by MVA-S(3P)-boosted regimens. By contrast, DREP-S/DREP-S elicited unexpectedly low anti-S IgG binding antibody titers, in contrast to our previous work where two doses of DREP-S delivered by intradermal electroporation induced mean anti-S IgG titers of 10^5^ in C57BL/6 mice 7 days post-boost^11^. The discrepancy likely reflects the route and delivery method of DREP-S: in the present study DREP-S was given intramuscularly without electroporation, which is known to reduce plasmid uptake/antigen expression and thereby antibody responses^6^.

Regarding the functional quality of the humoral responses induced, we first evaluated the neutralizing capacity of the sera obtained from immunized mice. In line with the anti-S IgG binding antibodies induced, neutralizing responses were robust and durable, but variant-specific. MVA-S(3P_Wuhan_) boosted neutralization against ancestral strains such as Wuhan and Beta at 10 days post-boost, although by six months post-boost, neutralizing activity persisted only against the Wuhan strain. Conversely, MVA-S(3P_XBB.1.5_) boosted neutralization against Omicron subvariants BA.1, BA.5, XBB.1.5 and XBB.1.16 at 10 days post-boost, with sustained activity at six months limited to XBB.1.5 and XBB.1.16, and with no neutralization of ancestral strains Wuhan and Beta.

Although traditional vaccine evaluation has primarily focused on neutralizing antibodies, accumulating evidence highlights the essential contribution of Fc-mediated effector functions in achieving durable and effective immunity^35^. Indeed, we previous demonstrated that the presence of neutralizing antibodies is not strictly required to confer in vivo protection^22^. For this reason, we further assessed Fc-dependent antibody functions, including ADCP, ADCD, and ADNKA, and we showed that MVA-S(3P)-based booster vaccinations consistently induced superior and longer-lasting Fc-mediated responses beyond neutralization. In general, the three groups of mice that received MVA-S(3P) as a booster displayed highly comparable ADCP, ADCD, and ADNKA functional profiles against Wuhan and XBB.1.5 S proteins. Over time, phagocytic capacity increased between day 38 and day 206 (10 days and 6 months post-boost, respectively), complement deposition remained largely stable, and NK cell activation showed a modest declined. These observations likely reflect the functional maturation and fine-tuning of the antibody response during the memory phase. Together, these findings also highlight the ability of MVA-S(3P) boosters to induce strong and durable Fc-dependent effector functions, and demonstrate that DREP, even when delivered suboptimally after intramuscular injection, serves as an effective prime. In the context of COVID-19 vaccination, it has been reported that administration of a third dose of an mRNA vaccine elicited impaired Fc-mediated effector functions in vitro, which persisted over time^36^. More recently, it has been reported that regimens including an S protein-based COVID-19 vaccine, either alone or as a booster following mRNA priming immunization, induced higher Fcγ effector activity compared with mRNA-only regimens^37^. These findings underscore the potential advantages of alternative vaccine platforms, such as DREP and MVA, in promoting durable Fc-mediated antibody functions and strengthening long-term protective immunity.

The quality of the B-cell response following vaccination is a critical determinant of both the durability and breadth of protective immunity^38^. In our study, B-cell phenotyping demonstrated the superior ability of MVA-S(3P) boosters to promote the expansion of GC B cells and swMBCs, two key indicators of antibody affinity maturation and long-term humoral immunity. These effects were already evident shortly after boosting in draining lymph nodes and were maintained at six months post-boost in the spleen, underscoring the capacity of MVA-S(3P) boosters to establish stable and durable B cell memory. During GC B cell activation, CD4⁺ Tfh cells provide essential cytokines and co-stimulation signals that drive B cell maturation and class switching^39^. In our study, the analysis of CD4⁺ Tfh cells revealed that at 10 days post-boost, the MVA-S/MVA-S(3P_Wuhan_) regimen was more effective at promoting expansion of the Tfh cell compartment; however, this expansion did not translate into effective S-specific functionality. At six months post-boost, all groups exhibited functional maturation of the CD4⁺ Tfh cell response, with those regimens receiving MVA-S(3P) as a booster showing the highest magnitudes of CXCR5⁺PD-1⁺ CD4⁺ T cells with a S-specific Th1-like functional profile (co-expressing CD154 and IFN-γ), which are instrumental in supporting GC reactions and high-quality antibody production^39^. Notably, these Tfh cell features persisted at late time points post-boost, indicating the generation of long-lived CD4⁺ Tfh cell memory.

Extensive research has shown that while antibodies are crucial for preventing SARS-CoV-2 infection, robust and durable CD4^+^ and CD8^+^ T-cell responses, largely preserved across variants, are essential for reducing disease severity and maintaining long-term immunity after vaccination^40,41^. In our study, S-specific CD4^+^ and CD8^+^ T-cell responses were significantly enhanced by MVA-S(3P)-boosted regimens compared with homologous DREP-S/DREP-S at both the adaptive (day 38; 10 days post-boost) and memory (day 206; 6 months post-boost) phases. Notably, at day 38 the heterologous DREP-S/MVA-S(3P_XBB.1.5_) and DREP-S/MVA-S(3P_Wuhan_) regimens elicited the strongest S-specific CD4⁺ and CD8⁺ T-cell responses, exceeding those induced by the homologous MVA-S/MVA-S(3P_Wuhan_) group. These findings are consistent with prior work from our groups showing that DREP/MVA prime/boost combinations synergistically enhance T-cellular immune responses across pathogens, including HIV^6^, CHIKV^3,4,10^, EBOV^9^, and HCV^8^. Although the overall magnitude of S-specific CD8⁺ T-cell responses declined over time, MVA-S(3P)-boosted groups maintained higher S-specific CD8⁺ T-cell frequencies. Importantly, MVA-S(3P)-boosted regimens induced highly polyfunctional S-specific CD4⁺ and CD8⁺ T cells, capable of cytokine secretion (IFN-γ, TNF-α, IL-2) and degranulation (CD107a expression), immune features associated with superior viral control^40,41^. Moreover, MVA-S(3P)-boosters clearly outperformed homologous DREP-S/DREP-S vaccination by eliciting high frequencies of polyfunctional S-specific CD4⁺ and CD8⁺ T cells that persisted for at least six months post-boost. The presence of polyfunctional T cells is a hallmark of protective immunity in both animal and human studies^42,43^. Moreover, the predominance of T effector memory cells (CD62L⁻, CD127⁺) observed in the MVA-S(3P)-boosted groups in the adaptive and memory phases may be particularly relevant for long-term protection, as these cells preferentially reside in peripheral tissues and can mount rapid and robust responses upon re-exposure to antigen^44^. Collectively, our results demonstrate that MVA-S(3P)-boosted groups induced potent, polyfunctional, and durable S-specific CD4⁺ and CD8⁺ T-cell responses with a predominantly effector memory phenotype.

While the apparent limitation of this study is the absence of results showing efficacy of DREP and MVA immunizations against SARS-CoV-2 infection, it should be pointed out that we have previously demonstrated in various preclinical studies the high efficacy of MVA-S vectors against SARS-CoV-2 in different animal models, including mice, hamsters and non-human primates^18–27^. For DREP-S, markers of immune efficacy against SARS-CoV-2 have also been provided^11^. Our interest here was to define in depth the SARS-CoV-2 specific cellular and humoral immune responses induced with time after DREP and MVA vaccination in mice, delivered either alone or in combination. In addition, all data presented here were generated from a single in vivo experiment, and pooled samples were used in selected assays to enable functional analyses. While these experimental choices may limit the assessment of inter-experimental variability, the relatively large number of animals per group, the longitudinal design, and the consistency of the results across multiple independent immunological readouts support the robustness of the findings. Nevertheless, future studies could be done in different animal models (hamsters and/or macaques) to further validate and extend these results.

Overall, our findings demonstrate that MVA-S(3P) boosters enhance humoral and cellular immune responses and improve their functional quality and durability, reinforcing their value in prime/boost vaccination strategies to optimize both humoral and cellular immunity against SARS-CoV-2 and its variants. In addition, we also provided important insights on the nature and role of immune cell populations differentially induced following DREP and MVA vaccinations. These novel findings position MVA as a robust and versatile booster platform for next-generation vaccines aimed at eliciting strong, broad, cross-reactive, and durable protective immune responses. Moreover, our study adds to the growing body of evidence highlighting heterologous DREP/MVA regimens as a promising approach to strengthen preparedness against current and future emerging infectious diseases.

## Materials and methods

### Animals and ethics statement

Female C57BL/6OlaHsd mice (6–8 weeks old) were obtained from Envigo Laboratories and housed under specific pathogen-free conditions in the biosafety level 2 (BSL-2) animal facility of the Centro Nacional de Biotecnología (CNB-CSIC, Madrid, Spain). All animal procedures were approved by the Ethical Committees of Animal Experimentation (CEEA) of CSIC and CNB-CSIC, and authorized by the Division of Animal Protection of the Comunidad de Madrid (PROEX 169.4/20). Animal experiments were conducted in accordance with Spanish (Royal Decree (RD) 53/2013) and European (Directive 2010/63/EU) regulations on animal welfare. The animal studies are reported in accordance with ARRIVE guidelines.

### Cells

Vero E6 cells (ATCC CRL-1586) were expanded in Dulbecco’s modified Eagle’s medium (DMEM; Gibco-Life Technologies) containing 10% heat-inactivated fetal bovine serum (FBS; Gibco-Life Technologies), HEPES buffer (10 mM; Gibco-Life Technologies), non-essential amino acids (0.1 mM; Sigma-Aldrich), and a standard penicillin/streptomycin mix (100 U/mL penicillin, 100 mg/mL streptomycin; Sigma-Aldrich). Vero/TMPRSS2 cells (derived from Vero E6 and stably expressing the TMPRSS2 protease under G418 selection) were maintained in DMEM containing 10% FBS, 10 mM HEPES, 0.1 mM non-essential amino acids, penicillin/streptomycin (100 U/mL penicillin, 100 mg/mL streptomycin), and the selective agent Geneticin (1 mg/mL, Merck-Life Sciences). The human monocytic line THP-1 (ATCC TIB-202) was cultured in RPMI-1640 medium (Sigma-Aldrich) supplemented with 10% FBS, L-glutamine (4 mM; Corning), 5% HEPES buffer (50 mM, pH 7.2; Corning), 5% penicillin/streptomycin (50 µg/mL; Corning), and 0.5% 2-mercaptoethanol (275 µM; Gibco-Life Technologies). All cell types were kept at 37 °C in a humidified 5% CO₂ atmosphere. Cell handling procedures were based on previously established protocols^22^.

### SARS-CoV-2 viruses

Several SARS-CoV-2 isolates were employed for the neutralization experiments. The MAD6 strain, corresponding to the ancestral Wuhan variant harboring the D614G substitution in the S protein, was obtained from Dr. José M. Honrubia and Prof. Luis Enjuanes (CNB-CSIC, Madrid)^45^. The B.1.351 (Beta) variant (hCoV-19/France/PDL-IPP01065i/2021) was acquired through the European Virus Archive-Global (EVAg) initiative, supplied by the National Reference Centre for Respiratory Viruses at Institut Pasteur (Paris, France). The Omicron BA.1 isolate (B.1.1.529; hCoV-19/Belgium/rega-20174/2021, EPI_ISL_6794907) was provided by Prof. Piet Maes (KU Leuven) via Dr. Robbert Boudewijns and Dr. Kai Dallmeier (KU Leuven, Belgium). Additional Omicron subvariants, including BA.5 (EPI_ISL_13424827), XBB.1.5 (EPI_ISL_16939528), and XBB.1.16 (EPI_ISL_17535655), were obtained from Prof. Rafael Delgado (Hospital Universitario 12 de Octubre, Madrid, Spain). Virus stocks were expanded in Vero/TMPRSS2 cells infected at low multiplicity of infection (MOI 0.001 plaque-forming units (PFUs)/cell). Culture supernatants were collected 72 h after inoculation, clarified by centrifugation, aliquoted, and stored at − 80 °C until use. Infectious titers were quantified in Vero E6 cells using plaque assays or median tissue culture infectious dose (TCID₅₀) determinations following established procedures^21,22^.

### DREP- and MVA-based vaccine candidates

Two different vector-based vaccination platforms were evaluated: a DNA-launched self-amplifying RNA replicon (DREP) and several modified vaccinia virus Ankara (MVA) constructs. The DREP-S candidate encoded the native full-length, non-stabilized SARS-CoV-2 S protein from the Wuhan strain, as previously described^11^. The recombinant MVAs included vectors expressing: (i) the native full-length S protein from the Wuhan strain (MVA-S), (ii) a Wuhan-derived prefusion-stabilized S protein carrying 3-proline modifications in S2 (MVA-S(3P_Wuhan_)), and (iii) an analogous prefusion-stabilized S antigen from the Omicron XBB.1.5 lineage (MVA-S(3P_XBB.1.5_)). MVA-S and MVA-S(3P_Wuhan_) have been characterized in earlier studies^18,21^, while the construction and validation of MVA-S(3P_XBB.1.5_) will be reported separately (Pérez et al., manuscript in preparation). All recombinant MVAs were expanded in permissive DF-1 cells, subsequently purified by sucrose-cushion ultracentrifugation, and viral titers were determined using an immunostaining-based plaque assay, as previously described^46^. Each stock was verified to be free of mycoplasma, bacterial, and fungal contamination using standard microbiological screening approaches. These procedures follow established protocols for MVA vector production^21,22^.

### Mouse vaccination protocol

Groups of female C57BL/6 mice (*n* = 12 per group, 6–8 weeks-old) were immunized with DREP-S and MVA-S(3P) vaccine candidates following homologous (DREP-S/DREP-S and MVA-S/MVA-S(3P_Wuhan_)) or heterologous (DREP-S/MVA-S(3P_Wuhan_) and DREP-S/MVA-S(3P_XBB.1.5_)) prime/boost immunization regimens, to evaluate the magnitude, breadth, and durability of the immunogenicity of the different vaccination regimens. Mice were immunized intramuscularly in both hind legs with 50 µg of DREP-S (in 100 µL PBS; 50 µL/leg) or 1 × 10⁷ PFUs of MVA-S (in 100 µL PBS; 50 µL/leg). Booster doses were administered on day 28 with homologous (DREP-S or MVA-S(3P_Wuhan_)) or heterologous (DREP-S/MVA-S(3P_Wuhan_) or DREP-S/MVA-S(3P_XBB.1.5_)) regimens. PBS-inoculated mice served as a control group. At days 38 (10 days post-boost) and 206 (6 months post-boost), 6 mice per group were sacrificed using carbon dioxide (CO_2_). Blood from each individual mouse was collected by cardiac puncture and processed to obtain serum samples to analyze SARS-CoV-2-specific humoral immune responses. On the other hand, inguinal lymph nodes and/or spleens were collected, pooled and processed to measure SARS-CoV-2-specific cellular immune responses. Additional blood samples were collected at days 14, 66, 94, 122, 150, and 178 by submandibular bleeding. Blood was incubated at 37 °C for 1 h, maintained at 4 °C overnight, and centrifuged at 3,600 rpm for 20 min at 4 °C to obtain serum samples. Serum was heat-inactivated (56 °C, 30 min) and stored at -20 °C until analysis of humoral immune responses. No adverse effects were observed in immunized mice at any timepoint. All data presented in this study derive from a single independent in vivo experiment, in which mice were longitudinally followed and sampled at defined time points.

### Serological analysis (ELISA)

Serum antibody responses against SARS-CoV-2 S protein were assessed by ELISA. Individual or pooled serum samples were analyzed for total IgG, IgG1, and IgG2c isotypes, as previously described^20^. For selected analyses, pooled sera were used to ensure sufficient sample volume and to obtain representative group-level measurements. High-binding M96 microplates were coated overnight with 2 µg/mL of the corresponding recombinant S antigen, followed by blocking and incubation with serially diluted sera in duplicate. Antigen-bound antibodies were detected using horseradish peroxidase (HRP)-conjugated anti-mouse IgG, IgG1, or IgG2c secondary antibodies (diluted 1:1000 in PBS 1X- 1% milk-0.1%Tween20). Reactions were developed with 3,3’,5,5’-Tetramethylbenzidine (TMB) substrate, and absorbance was measured at 450 nm. Endpoint titers were calculated as the highest serum dilution yielding an optical density exceeding three times that of naïve controls; values below the detection threshold were assigned as half of that limit. The ELISA antigens consisted of soluble S proteins derived from the ancestral Wuhan strain (GenBank MN908947.3), the Beta variant (EPI_ISL_712096), and Omicron subvariants BA.1 (OL672836.1), BA.5 (ON249995.1), and XBB.1.5 (OP790748.1). Recombinant expression plasmids, protein production in mammalian systems, and purification from culture supernatants were performed following established procedures^18,20^.

### Live SARS-CoV-2 virus neutralization assay

The neutralizing activity of sera collected from immunized mice was evaluated in a BSL-3 facility (CNB-CSIC) using a live-virus microneutralization (MNT) assay, as previously described^22^. Heat-inactivated individual or pooled serum samples were serially diluted in DMEM supplemented with 2% FBS and mixed 1:1 with 100 TCID₅₀ of the indicated SARS-CoV-2 isolates, including the ancestral Wuhan (MAD6), Beta, and Omicron BA.1, BA.5, XBB.1.5, and XBB.1.16 variants. When indicated, pooled sera were used for variant breadth analyses. After a 1-hour incubation at 37 °C, the virus–serum mixtures were transferred in triplicate onto Vero E6 cell monolayers (1.3 × 10⁴ cells per well) seeded in 96-well plates. Following a 72-hour infection period, cells were fixed and stained with 5% crystal violet (Sigma-Aldrich) to assess cytopathic effect. Stained monolayers were subsequently solubilized with 1% SDS (Sigma-Aldrich), and absorbance at 570 nm was recorded to determine the percentage of neutralization. To obtain the 50% neutralization titers (NT_50_), half maximal effective concentration (EC_50_) and corresponding 95% confidence intervals were obtained by fitting the data to a nonlinear regression curve (agonist concentration versus normalized response) using GraphPad Prism v10.3.1.

### Antibody-dependent cellular phagocytosis (ADCP)

The ability of antibodies present in pooled sera from immunized mice to mediate ADCP was evaluated as follows. Pooled sera were used to ensure sufficient sample volume and to obtain representative functional measurements. SARS-CoV-2 S proteins from the Wuhan strain or the Omicron XBB.1.5 variant were covalently coupled to fluorescent beads (1 μm Red Carboxylate-Modified FluoSpheres; Invitrogen), following the manufacturer´s protocol. Bead–antigen conjugates were incubated with serum samples diluted 1:10 in PBS to allow immune complex formation (2 h at 37 °C), and washed with PBS. THP-1 cells were then added at 1 × 10^5^ cells/mL and incubated overnight at 37 °C with 5% CO_2_. The uptake of immune complex–coated beads by THP-1 cells was quantified by flow cytometry using a CytoFLEX cytometer (Beckman Coulter Life Sciences). All samples were assayed in triplicate (see Supplementary Fig. 1).

### Antibody-dependent complement deposition (ADCD)

To assess the capacity of antibodies elicited by the vaccines to promote complement activation, ADCD assays were performed using fluorescent antigen-coated beads. Red Carboxylate-Modified FluoSpheres (1 μm; Invitrogen) were covalently coupled to SARS-CoV-2 S proteins from the Wuhan strain or the Omicron XBB.1.5 variant, and immune complexes were generated as described for the ADCP assay. After washing, lyophilized guinea pig complement (Sigma-Aldrich), diluted 1:25 in RPMI-10%FBS, was added and samples were incubated for 20 min at 37 °C. Complement C3 deposition on bead surfaces was detected by staining with FITC-conjugated anti-guinea pig C3 (Invitrogen) and quantified by flow cytometry using a CytoFLEX cytometer (Beckman Coulter Life Sciences). Assays were performed using pooled sera to ensure sufficient sample volume and representative group-level measurements.

### Antibody-dependent natural killer cell activation (ADNKA)

To evaluate the ability of vaccine-induced antibodies to trigger NK cell effector functions, ADNKA assays were performed as previously described^47^. Pooled sera were used for these analyses to ensure sufficient sample volume and to obtain representative functional responses. Thermo NUNC MaxiSorp 96-well plates were coated overnight at 4 °C with SARS-CoV-2 S proteins (Wuhan or Omicron XBB.1.5) at 3 µg/mL in BBS. After three washes with PBS-Tween, wells were blocked in PBS-1% casein (PBS-C) overnight at 4 °C. Pooled serum samples diluted in PBS-C were then added and incubated for 2 h at room temperature. Mouse NK cells were isolated from splenocytes using the NK Cell Isolation Kit (Miltenyi Biotec; negative selection) and expanded in complete RPMI supplemented with 1% non-essential amino acids (Gibco), 1 mM MEM sodium pyruvate (Gibco), 50 µM β-mercaptoethanol, 1000 U/mL mouse IL-2, and 50 ng/mL mouse IL-15 for 7 days at 37 °C and 5% CO_2_. At days 3 and 5 NK cells were re-stimulated with IL-2 and IL-15. Following serum incubation, plates were washed with PBS-Tween and PBS, and NK cells were added at 5 × 10⁴ cells/well in 200 µl of RPMI-10% FBS supplemented with monensin (1X) and anti-CD107a-FITC (1 µg/mL). After 5 h of incubation at 37 °C and 5% CO_2_, cells were stained with anti-CD3-PE-CF594 and NK1.1-PE antibodies in PBA for 30 min, washed with PBS, fixed/permeabilized with BD Cytofix/Cytoperm™ Fixation/Permeabilization Kit, and stained intracellularly with anti-IFN-γ-PE-Cy7 and anti-TNF-α-APC. Samples were acquired using a CytoFLEX cytometer (Beckman Coulter Life Sciences). Data are shown in Supplementary Fig. 2.

### Memory B cell assay

Draining lymph nodes, extracted at 10 days post-boost (day 38), and spleens extracted 6 months post-boost (day 206) from each immunized mouse were pooled per group, processed mechanically, blood-cell depleted, and filtered through 40-µm cell strainers until single-cell suspensions were obtained. Then, 2 × 10^6^ live cells were seeded into 96-well V-bottom plates and stained with B cell markers (CD3^−^, B220^+^, CD19^+^) to define GC B cells (GL7⁺, CD38⁻), memory B cells (GL7⁻, CD38⁺), and isotype-switched memory B cells (IgD⁻, IgM⁻). Cells were acquired on a CytoFLEX cytometer (Beckman Coulter Life Sciences) and analysis was performed using FlowJo software version 10.4.2 (Tree Star).

### Intracellular cytokine staining (ICS) assay

SARS-CoV-2 S-specific CD4^+^ Tfh responses were measured by ICS following stimulation of 2 × 10^6^ pooled splenocytes per group with SARS-CoV-2 S peptide pools (JPT Peptide Technologies; 1 µg/mL, spanning the entire S protein from the Wuhan strain as consecutive 15-mers overlapping by 11 amino acids) plus Wuhan S protein in the presence of anti-CD154 (CD40L)–PE (BD Biosciences) for 6 h. After 2 h cytokine secretion was inhibited by adding brefeldin A (BD Biosciences) and monensin (BD Biosciences). The following fluorochrome-conjugated antibodies were used: CD4-Alexa Fluor 700, CD8-V500, PD1 (CD279)-APC-efluor780, and CXCR5-PE-CF594 for phenotypic analyses, and CD154-PE, and IFN-γ-PE-Cy7 for functional analyses. All antibodies were from BD Biosciences.

Similarly, for SARS-CoV-2 S-specific CD4^+^ and CD8^+^ T-cell responses, 2 × 10^6^ pooled splenocytes per group were stimulated for 6 h with the same SARS-CoV-2 S peptide pools in the presence of brefeldin A (BD Biosciences), monensin (BD Biosciences) and 1 µg/mL anti-CD107a FITC. Then, the magnitude, polyfunctionality and memory phenotype of SARS-CoV-2 S-specific CD4^+^ and CD8^+^ T cells expressing CD107a, and/or secreting IFN-γ, and/or TNF-α, and/or IL-2 were analyzed by an ICS assay, as previously described^18^. Cells were stained for surface markers (CD3, CD4, CD8, CD127, CD62L, CD107a) and cytokines (IFN-γ, IL-2, TNF-α).

Cells were acquired with a CytoFLEX cytometer (Beckman Coulter Life Sciences), and data were analyzed using FlowJo software version 10.4.2 (Tree Star), as previously described^18^.

### Statistical analysis

All graphs, calculations, and statistical analyses were performed using GraphPad Prism software. A mixed-effects model with Geisser-Greenhouse correction, followed by Tukey’s multiple comparisons test on transformed data, was used to analyze anti-S (Wuhan and XBB.1.5) IgG titers and live virus neutralizing antibody titers (NT_50_) from individual mouse serum samples. Each mouse was considered an individual biological replicate unless otherwise indicated. An ordinary two-way ANOVA followed by Tukey’s multiple comparisons test on transformed data was used to analyze IgG titers against S proteins from SARS-CoV-2 variants and NT_50_ values from pooled serum samples. Group comparisons for IgG2c/IgG1 ratios, % phagocytosis, % C3 deposition and NK cell activation (all from pooled serum samples), and frequencies of B cell and T helper cell subpopulations, as well as ICS-assessed S-specific CD4⁺ and CD8⁺ T cells producing cytokines (all from pooled splenocytes or pooled draining lymph node cells), were performed using an ordinary two-way ANOVA with Tukey’s post hoc test. For statistical analysis of the frequency of CD4⁺ T cells expressing CD154 (CD40L) and producing IFN-γ following stimulation with SARS-CoV-2 S peptide pools (analyzed by ICS in pooled splenocytes), an ordinary one-way ANOVA followed by Tukey’s multiple comparisons test was used. Statistical significance is indicated as follows: *p < 0.033; **p < 0.002; ***p < 0.0002; ****p < 0.0001.

## Supplementary Information

Below is the link to the electronic supplementary material.

Supplementary Material 1.

## Data Availability

The datasets used and/or analysed during the current study available from the corresponding author on reasonable request.
