# Supplementary material for: Homologous MVA and heterologous DREP/MVA vaccine regimens induce robust and durable immune responses against SARS-CoV-2
Source: Sci Rep. 2026 Apr 9;16:16207. doi: 10.1038/s41598-026-46699-0 (PMC13201570; doi:10.1038/s41598-026-46699-0)
Supplement: Supplementary file 1 — Supplementary Material 1. [file 41598_2026_46699_MOESM1_ESM.pdf]

Supplementary information

Homologous MVA and heterologous DREP/MVA vaccine regimens induce robust and durable immune responses against SARS-CoV-2

Patricia Pérez [1,2], Gloria Esteso [1], María A. Noriega [1], Laura Perez Vidakovics [3], Peter Liljeström [3], Gerald M. McInerney [3], Mariano Esteban [1], and Juan García-Arriaza [1,2,*]

[1] Department of Molecular and Cellular Biology, Centro Nacional de Biotecnología (CNB), Consejo Superior de Investigaciones Científicas (CSIC), Madrid, Spain. [2] Centro de Investigación Biomédica en Red de Enfermedades Infecciosas (CIBERINFEC), Madrid, Spain. [3] Division of Virology and Immunology, Department of Microbiology, Tumor and Cell Biology, Karolinska Institutet, Stockholm, Sweden.

* Corresponding author: Juan García-Arriaza (jfgarcia@cnb.csic.es)

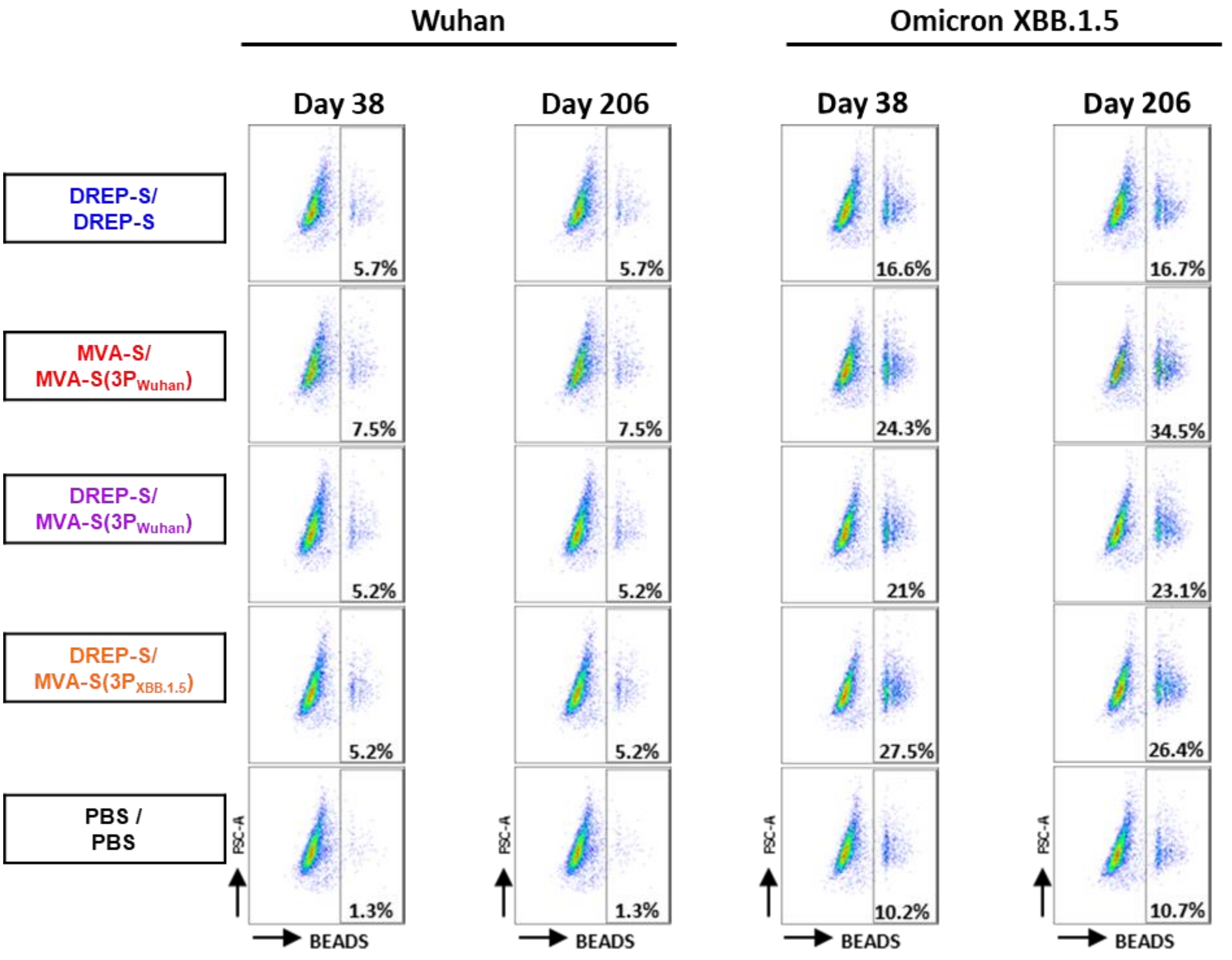

Supplementary Figure 1. Antibody-dependent cellular phagocytosis (ADCP) assay. Representative scatterplots from THP-1 cells incubated overnight with immune complexes formed by fluorescent beads coated with SARS-CoV-2 S protein (Wuhan or Omicron XBB.1.5) and sera from immunized mice. Plots show bead uptake by THP-1 induced by the pooled sera from different vaccination groups and the PBS control group at days 38 and 206. Scatterplots also illustrate the gating strategy used to quantify phagocytic activity shown in Figure 3A.

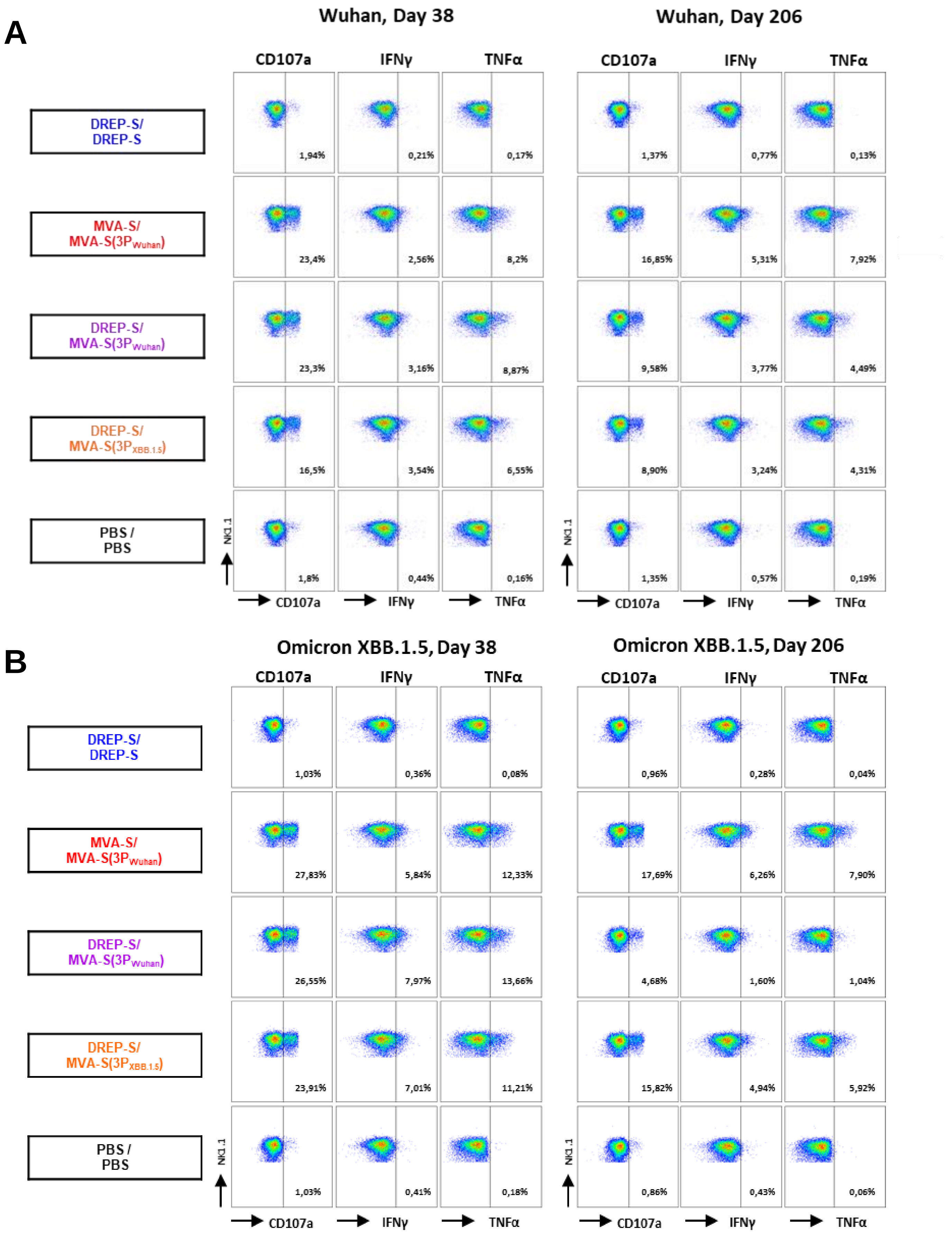

Supplementary Figure 2. Antibody-dependent natural killer cell activation (ADNKA) assay. Representative scatterplots of mouse spleen NK cells after stimulation with SARS-CoV-2 S protein Wuhan (A) or Omicron XBB.1.5 (B). Plots show NK cells expressing CD107a (degranulation marker), IFN-γ, and TNF-α after incubation with S, from Wuhan or Omicron XBB.1.5, in the presence of pooled sera from the different vaccination groups and the PBS control group at days 38 and 206. Scatterplots also illustrate the gating strategy used to quantify ADNKA summarized in Figure 3C.
